# Supplementary material for: Comparison of Drug-Related Problems in COVID-19 and Non-COVID-19 Patients Provided by a German Telepharmacy Service for Rural Intensive Care Units
Source: J Clin Med. 2023 Jul 18;12(14):4739. doi: 10.3390/jcm12144739 (PMC10380643; doi:10.3390/jcm12144739)
Supplement: Supplementary file 1 [file jcm-12-04739-s001.zip › jcm-2463095-supplementary.pdf]

Supplement

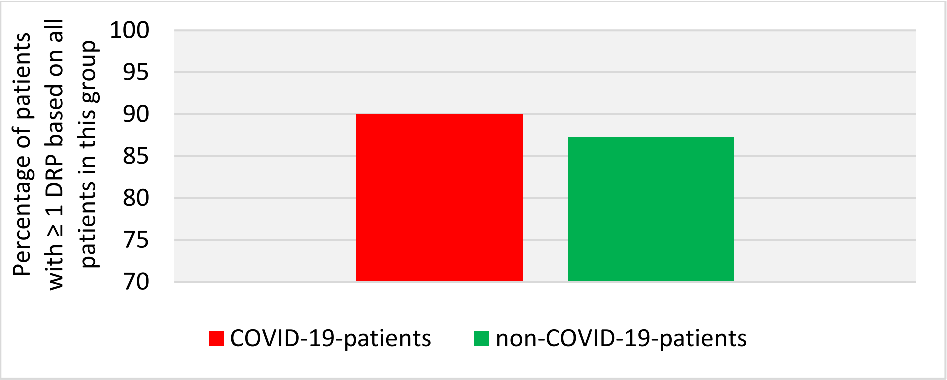

A

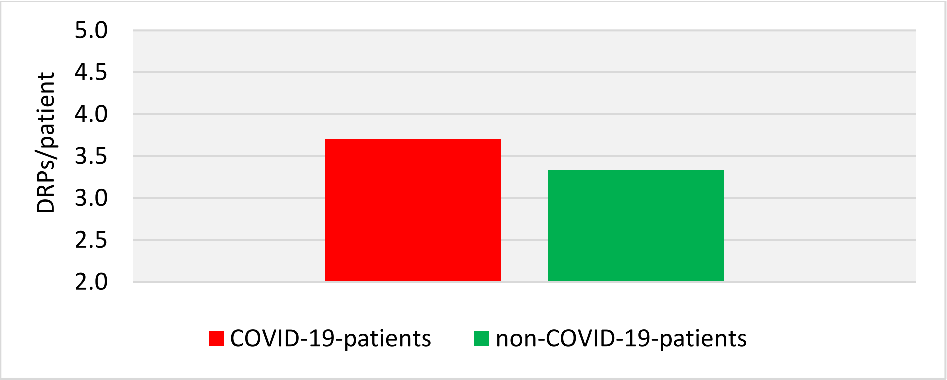

B

Figure S1. Percentages of patients with ≥1 DRP based on all patients and DRPs/patient-ratios in in COVID-19 and non-COVID-19 patients. Part A shows the percentages of patients with ≥1 DRP based on all patients in COVID-19 and non-COVID-19 patients. The difference is non-significantly different (COVID-19 patients: 172 of 191 patients suffered from ≥ 1 DRP, non-COVID-19 patients: 282 of 323 patients). Part B shows the DRPs/patient-ratios in in COVID-19 and non-COVID-19 patients (non-significantly different, COVID-19 patients: 707 DRPs in 191 patients, non-COVID-19 patients: 1077 DRPs in 323 patients).

DRP category; percentage of total DRPs	The three most prevalent drug classes with regard to total drug entries	Total drug entries (n, [%])	Drug entries for COVID-19 (n, [%])	Drug entries for NON-COVID-19 (n, [%])
(Clear) indication not (or no longer) given;<br>15.4% of DRPs [274/1784 DRPs]	1. Proton pump inhibitors (A02BC)	57 [20.7]	28 [21.9]	29 [19.7]
	2. Propulsives (A03FA)	50 [18.2]	18 [14.1]	32 [21.8]
	3. Preparations inhibiting uric acid production (M04AA)	13 [4.7]	6 [4.7]	7 [4.8]
(Clear) indication, but no drug prescribed; 13.2% of DRPs [235/1784 DRPs]	1. HMG CoA reductase inhibitors (C10AA)	33 [13.8]	13 [13.8]	20 [13.8]
	2. Proton pump inhibitors (A02BC)	31 [13.0]	17 [18.1]	14 [9.7]
	3. Peripheral opioid receptor antagonists (A06AH)	27 [11.3]	12 [12.8]	15 [10.3]
(Inappropriate) dose; 11.0% of DRPs [197/1784 DRPs]	1. Heparin group (B01AB)*	53 [27.3]	31 [38.8]<br><br>14x DI<br>17x DR	22 [19.3]<br><br>16x DI<br>6x DR
	2. Proton pump inhibitors (A02BC)*	43 [22.2]	8 [10.0]<br><br>2x DI<br>6x DR	35 [30.7]<br><br>2x DI<br>33x DR
	3. Osmotically acting laxatives (A06AD)	21 [10.8]	6 [7.5]<br><br>All DI	15 [13.2]<br><br>All DI
TDM not performed or not considered; 9.6% of DRPs [171/1784 DRPs]	1. Heparin group (B01AB)*	51 [29.5]	37 [46.3]	14 [15.1]
	2. Magnesium (A12CC)	37 [21.4]	15 [18.8]	22 [23.7]
	3. Digitalis glycosides (C01AA)*	24 [13.9]	6 [7.5]	18 [19.4]
Inappropriate or not most suitable drug in terms of indication; 8.2% of DRPs [146/1784 DRPs]	1. Heparin group (B01AB)	28 [19.2]	12 [20.3]<br><br>4x Heparin → LMWH<br>6x LMWH → Heparin<br>2x LMWH → Apixaban/ Phenprocoumon	16 [18.0]<br><br>11x Heparin → LMWH<br>5x LMWH → Heparin
	2. HMG CoA reductase inhibitors (C10AA)	18 [12.3]	8 [13.6]<br><br>All Simvastatin → Atorvastatin	10 [11.2]<br><br>All Simvastatin → Atorvastatin
	3. Sulfonamides, plain (C03CA)	17 [11.6]	5 [8.5]<br><br>All Furosemide → Torasemide	12 [13.5]<br><br>11x Furosemide → Torasemide<br>1x Torasemide → Furosemide

Table S1. Overview for the five most prevalent DRP categories and associated three most common drug classes (please refer to Figure 2 in the main publication). DI = dose increase, DR = dose reduction, LMWH = low molecular weight heparin, TDM = therapeutic drug monitoring. Asterisk indicates significant difference between COVID-19 and non-COVID-19 ($p < 0.05$).

Drug class	DRP topics	Number of DRPs
Glucocorticoids (H02AB, n = 22)	Enteral administration of dexamethasone (instead of intravenous, same dosage)	14
	Choice of corticoid agent and dosing for COVID-19 when already systemic therapy due to other reason (septic shock, ankylosing spondylitis)	4
	Control of side effects (blood sugar, deterioration of glaucoma)	2
	Termination of dexamethasone therapy after 10 days	1
	Choice of corticoid therapy in pregnancy	1
Interleukin inhibitors (L04AC, n = 3)	Assessment of indication and dosage for tocilizumab in a specific patient	3
Nucleosides and nucleotides excl. reverse transcriptase inhibitors (J05AB, n = 1)	Comparison of outcomes when 5 compared to 10 days therapy with remdesivir	1

Table S2. DRPs with COVID-19 specific medications (antiviral or immunosuppressive therapies).

	COVID-19 patients				NON-COVID-19 patients			
	patients	DRPs	DRPs specific for renal insufficiency	Patients with ≥ 1 DRP	patients	DRPs	DRPs specific for renal insufficiency	Patients with ≥ 1 DRP
eGFR ≥ 60, no RRT	104	299	0	88	151	387	0	122
eGFR 30-59, no RRT	22	83	11	21	51	141	14	46
eGFR < 30, no RRT	15	54	12	14	39	112	34	37
iHD	1	1	1	1	3	16	2	3
PiRRT/ CVVH (DF)	8	14	0	7	12	29	4	9

Table S3. Number of patients, DRPs, DRPs specific for renal insufficiency, and patients with at least one DRP separated to the groups COVID-19 patients and non-COVID-19 patients. Considered for inclusion into this table were patients, that did not change the group of renal insufficiency during the consultations (79%, 406 of 514 patients). CVVH (DF) = continuous venovenous hemodialysis/ hemodiafiltration; eGFR = estimated glomerular filtration rate (CKD-EPI); iHD = intermittent hemodialysis; PiRRT = prolonged intermittent kidney replacement therapy; RRT = renal replacement therapy.

Drug class (ATC-Code)	Total (n)	COV (n)	NCOV (n)	Topics of the DRPs
Heparin group (B01AB)	29	15	14	• Enoxaparin in therapeutic-dose: monitoring of anti-Xa in renal insufficiency (n = 13)<br>• Enoxaparin in eGFR < 30ml/min/1.73m²: not recommended according to SmPC, change to heparin (n = 8)<br>• No anticoagulation prescribed, when indicated (n = 2)<br>• Consultation for choosing most suitable drug in specific patients (n = 2)<br>• Enoxaparin: dose adjustment due to accumulation risk (n = 1)<br>• Enoxaparin in therapeutic dose: 12-hours dosing interval instead of 24-hours recommended (n = 1)<br>• Heparin: therapeutic-dose indicated (n = 1)<br>• Heparin: subcutaneous application in obese class III and severe renal insufficiency: change to infusion pump with aPTT-monitoring (n = 1)
Carbapenems (J01DH)	16	5	11	All DRPs were associated with meropenem<br>• Dose reduction due to renal function (n = 9)<br>• Dose increase recommended due to renal function and/or effectivity of RRT (n = 7)
Propulsives (A03FA)	15	2	13	All DRPs were associated with Metoclopramide<br>• Dose reduction due to eGFR< 30 or < 60ml/min/1.73m² according to SmPC (n = 11)<br>• Recommendation to choose metoclopramide in reduced dosing for gastroparesis (n = 3)<br>• Recommendation to change metoclopramide to domperidone due to possible accumulation in renal insufficiency and central antidopaminergic effects (n = 1)
HMG CoA reductase inhibitors (C10AA)	10	5	5	• Change from simvastatin to atorvastatin (due to SmPC simvastatin doses above 10mg/day should be used with caution, n = 8)<br>• Statin indicated: due to renal insufficiency choose atorvastatin (n = 1)<br>• Keep simvastatin paused postoperatively, until eGFR is > 30ml/min/1.73m² (n = 1)
Combinations of penicillins, incl. beta-lactamase inhibitors (J01CR)	10	4	6	• Dose reduction due to renal insufficiency (n = 9, 8-times pipacillin/tazobactam, once ampicillin/sulbactam)<br>• Continuation of piperacillin/tazobactam 3x/day 4.5g at GFR < 30 due to BMI 40 kg/m² (n = 1)
Thiazides, plain (C03AA)	6	1	5	All DRPs associated with hydrochlorothiazide (HCT)<br>• Due to SmPC use of HCT contraindicated in eGFR < 30 ml/min/1.73m² (lack of efficacy, n = 4)<br>• Choose HCT for sequential nephron blockade in high doses of loop diuretics (n = 2)
ACE inhibitors, plain (C09AA)	5	1	4	• Dose reduction of ramipril in eGFR < 30ml/min/1.73m² and/or hemodialysis (n = 4)<br>• Lisinopril contraindicated in eGFR < 30ml/min/1.73m² (n = 1)

Natural opium alkaloids (N02AA)	5	2	3	• Change morphine or oxycodone to hydromorphone / choose hydromorphone in chronic pain - due to eGFR < 30 ml/min/1.73m² (n = 4)<br>• Morphine use in RRT favored by physician: watch of potential symptoms of glucuronide accumulation (n = 1)
Glycopeptide antibacterials (J01XA)	4	1	3	• Vancomycin level monitoring daily or every other day in eGFR < 30 or < 60 ml/min/1.73m² (n = 4)
Pyrazolones (N02BB)	4		4	• Discontinue metamizole (NSAID) in eGFR < 60 ml/min/1.73m² (n = 4)
Other antiepileptics (N03AX)	4	3	1	• Pregabalin: dose reduction due to eGFR < 30 ml/min/1.73m² (n = 2)<br>• Levetiracetam: dose reduction due to eGFR < 30 ml/min/1.73m² (n = 1)<br>• Levetiracetam: return from reduced dose to ambulant prescribed dose due to normalized renal function (n=1)
Fluoroquinolones (J01MA)	4	2	2	• Levofloxacin: dose reduction due to eGFR < 30 ml/min/1.73m² (n = 2)<br>• Ciprofloxacin: dose reduction due to eGFR < 30 ml/min/1.73m² (n = 2)
Dipeptidyl peptidase 4 (DPP-4) inhibitors (A10BH)	3	2	1	• Sitagliptin: dose reduction due to eGFR < 60ml/min/1.73m² (n = 3)
Aldosterone antagonists (C03DA)	3		3	All DRPs associated with spironolactone<br>• Contraindicated in eGFR < 30 ml/min/1.73m² (n = 2)<br>• Adverse event: hyperkalemia potentially due to outpatient use in eGFR 30-40 ml/min/1.73m² - blood pressure sufficient, keep paused (n = 1)
Beta-lactamase resistant penicillins (J01CF)	3		3	• Flucloxacillin: no dose reduction in eGFR > 10ml/min/1.73m² - risk of underdosing (n = 3)
Miscellaneous	16	6	10	• Edoxaban: dose reduction in eGFR < 30 ml/min/1.73m² (n = 2)<br>• Change torasemide in eGFR < 30 ml/min/1.73m² to furosemide to better regulate intravasal volume (n = 1)<br>• Change furosemide from regular applications to “on demand” (increasing creatinine and reduced urine volume, n = 1)<br>• Better prescribe pantoprazole instead of ranitidine for stress ulcer prophylaxis in a patient with intermittent hemodialysis (n = 1)<br>• Methylnaltrexone: dose reduction in eGFR < 30 ml/min/1.73m² (n = 1)<br>• Metformin: dose reduction in eGFR < 30 ml/min/1.73m² (n = 1)

				• Epoetin alfa application in chronic kidney disease: monitoring of serum iron recommended (n = 1)<br>• Digitoxin plasma level monitoring in eGFR < 10ml/min/1.73m² (n = 1)<br>• Adverse event: hyperkalemia potentially due to outpatient use of candesartan in eGFR 30-40 ml/min/1.73m² - blood pressure sufficient, keep paused (n = 1)<br>• Fluconazole: dosing recommendation for loading and maintenance dose in acute kidney injury (n = 1)<br>• Parecoxib application necessary due to physician: dose reduction in eGFR 30-40 ml/min/1.73m² recommended according to SmPC (n = 1)<br>• Patient with chronic pain: choose paracetamol as non-opioid analgesic in eGFR < 30ml/min/1.73m² (n = 1)<br>• Lithium intoxication due to acute kidney injury – monitor lithium plasma levels daily for one week (n = 1)<br>• Milnacipran: dose reduction recommended in eGFR < 30 ml/min/1.73m² (n = 1)<br>• Cetirizine contraindicated in eGFR < 30ml/min/1.73m² - stop medication (n = 1)

Table S4. Drugs and drug classes that were involved in DRPs that were related to the patients' renal insufficiency. All drug classes with n ≥3 DRPs are shown in detail, the remaining drugs are summarized in the category "miscellaneous". aPTT = activated partial thromboplastin time, eGFR = estimated glomerular filtration rate (CKD-EPI), NSAID = non-steroidal anti-inflammatory drug, SmPC = Summary of product characteristics.
